# Functional role of cancer stem cell like exosomes on survival and drug resistance behaviors of colorectal cancer cells

**DOI:** 10.1007/s12672-025-04295-0

**Published:** 2025-12-20

**Authors:** Elmira Gheytanchi, Marzieh Naseri, Feridoun Karimi-Busheri, Fatemeh Tajik, Faezeh Vakhshiteh, Roya Ghods, Zahra Madjd

**Affiliations:** 1 Oncopathology Research Center, Iran University of Medical Sciences, Tehran, Iran; 2 Department of Molecular Medicine, Faculty of Advanced Technologies in Medicine, Iran University of Medical sciences, Tehran, Iran; 3 Department of Developmental, Molecular and Chemical Biology, Tufts University School of Medicine, Boston, USA; 4 Department of Oncology, Cross Cancer Institute, Faculty of Medicine, University of Alberta, Edmonton, AB Canada; 5 Department of Surgery, University of California, Irvine Medical Center, Orange, CA USA

**Keywords:** Cancer stem cells (CSCs), Colorectal cancer (CRC), Drug resistance, Sphere formation, Exosome.

## Abstract

**Purpose:**

This study aimed to evaluate the potential impact of colorectal cancer stem cell exosomes (CRC CSCs-enriched exosomes/CSCs-EXOs) on drug resistance and cell proliferation of CRC tumor cells.

**Methods:**

CSCs were enriched from HT-29 cells and characterized by sequential sphere formation, real-time PCR analysis of key stemness genes, and CRC-CSCs markers. The gene expression related to ABC transporters was analyzed in HT-29, HT-29 CSCs, and Caco-2 cells. CSCs-EXOs and parental-EXOs were isolated and characterized from HT-29 cells. The gene expression related to ABC transporters was investigated in Caco-2 cells treated with CSCs-EXOs and parental-EXOs of HT-29 cells by real-time PCR. The survival rate of exosome-treated Caco-2 cells was also studied in the presence of 5-fluorouracil (5-FU) at the IC50 concentration using MTT assay.

**Results:**

Colonospheres were found to have the ability to form serial spheres, along with the upregulatation of the key stemness genes (*p-value* ≤ 0.05). The expression of CRC-CSCs markers significantly increased relative to their parental counterparts (*p-value* ≤ 0.05). Treatment of Caco2 cells with CSCs-EXOs and their parental-EXOs revealed a substantial elevation in expression of drug resistance genes relative to those treated with their parental-EXOs (*p-value* ≤ 0.0001). The combination treatment of cells with exosomes and 5-FU at the IC50 concentration led to a more pronounced decrease in cell viability in all groups compared to applying 5-FU at the same concentration.

**Conclusion:**

Our findings underscore the significance of targeting the CSCs-exosome axis as a prospective therapeutic strategy to overcome drug resistance. Upcoming studies ought to concentrate on exploring the molecular machinery of CSCs and tumor cells plasticity through the exosome-mediated functions.

**Supplementary Information:**

The online version contains supplementary material available at 10.1007/s12672-025-04295-0.

## Introduction

Anti-cancer therapies frequently cause tumor recurrence mainly due to the failure to eradicate and target the cancer stem cells (CSCs) population [1, 2]. CSCs are a unique subset of heterogeneous tumor cells notable for their capacity for self-renewal and potential for multi-lineage differentiation, drug resistance, and higher tumorigenicity. They are believed to be responsible for metastasis and tumor recurrence [3–5]. Accordingly, exploring the underlying molecular machineries linked to the CSCs phenotype is desired to develop innovative therapeuticstrategies [5, 6]. An increasing amount of evidence has shown that the CSCs fulfill their “stemness-related duties” and enhance the epithelial-mesenchymal transition (EMT) and drug resistance phenotypes through the release of some factors, including exosomes [2–4]. Exosomes are membranous nanovesicles (30–150 nm) that carry cellular biomaterials, particularly proteins and RNAs (microRNAs and lncRNAs) and play a role in intracellular communication [2–4, 7]. A variety of studies have underscored the significance of exosomes in the progression of cancer [8–13], replicative immortality, angiogenesis, drug resistance [14], metastasis [4, 6, 15, 16], and development of pre-metastatic niche [17]. Hence, unraveling the precise molecular mechanisms of these nanovesicles in cancer could lead to the discovery of innovative approaches in the fight against cancer [3]. Increasing evidence has supported the dual role of exosomes in cancer through the inhibition or progression of the proliferation rates of tumor cells and their responses to radio- and chemotherapy [3, 5, 6, 8, 18]. Derived microvesicles from CD105^+^ cancer stem cells in human renal cell carcinoma induce pro-angiogenic effects, including endothelial cell proliferation, invasion, resistance to apoptosis, and development of a pre-metastatic lung niche [19]. The stemness and stem-like properties of non-CSCs population could be fortified after delivery of CSCs-derived extracellular vesicles (EVs), including exosomes containing proteins and markers indicative of stemness, such as Notch1, CD133, and CD44 [20–24]. Non-glioblastoma stem cells (non-GSCs) could be reprogrammed into GSCs with enhanced tumorigenicity through the exosomal Notch1 [20]. The exosomal cargo of esophageal squamous CSCs (ESCC) and clear cell renal CSCs (CCRCC) accelerated cell proliferation, EMT process, anti-apoptosis process, lung metastasis, and tumor cell invasion [16, 24]. Cell death and apoptosis have been induced in lung cancer through the high exosomal cargo of GAS5 [25]. In addition, the promotion or reduction of cancer cell proliferation has been supported by the findings of several studies [26–29]. Exosomes could affect cancer growth by modulating cell cycle, proliferation potential and apoptosis inducing activity through the exosomal contents [30]. Tumor exosomes (TEXs) could regulate or modulate the proportion of cells in G0 to G1 phase and mediate the expression levels of proteins that contribute to cell proliferation [9–13] or apoptosis [31] and trigger related signaling pathways [18, 32]. The results of molecular profiling showed that the exosomal cargo could regulate cell proliferation and apoptosis of ESCC [32, 33]. However, the putative role of CSCs-EXOs, as a subtype of tumor exosomes, remains an area of unmet research need. Given the biological features of CSCs-EXOs, investigating their effects and underlying mechanisms in cancer development and progression has far-reaching significance to maximize their benefit in cancer management. Moreover, current research efforts tend to explore the differences between CSCs-EXOs and TEXs [34]. Prostate and CRC CSCs-EXOs contain different cargoes compared to the TEXs from the bulk of the tumor [35, 36]. According to the crucial role of CSCs, it seems that CSCs-EXOs can alter tumor cell phenotype by transferring key stemness and drug resistance characteristics [3, 16, 19, 23, 35–37]. Previously, we have reported that the expression levels of genes associated with drug resistance (ABC transporters) were considerably higher in HT-29 CSCs-enriched spheroids relative to their parental HT-29 cells, as well as parental Caco-2 cells [38]. Then, the hypothesis was raised that CSCs exosomes are capable of inducing drug resistance gene expression even in a different colorectal cancer cell line. The present research intends to provide a standpoint on the potential impact of HT-29 CSCs-EXOs in the induction of drug resistance and cell proliferation of tumor cells as their attributed features compared to the parental TEXs. The insights gained from this research would present a new perspective to support upcoming studies in the context of translational medicine for identifying new approaches to manage CRC.

## Materials and methods

### CSCs isolation, enrichment and characterization

#### Spheroid culture using hanging droplets

The human colon adenocarcinoma cell lines; Caco-2 (IBRC C10094) and HT-29 (IBRC C10097) were obtained from the Iranian Biological Resource Center (IBRC, Tehran, Iran), and were maintained under standardized culture conditions in accordance with the supplier’s protocol [38]. Briefly, they were cultured in DMEM/high glucose (Gibco, Germany) supplemented with 10% FBS (Gibco, Germany), 2 mM L-glutamine (Gibco, Germany), 100 U/mL penicillin, and 100 µg/mL streptomycin antibiotics (Biowest, France) at 37 °C, 5% CO_2_ humidified incubator [38]. HT-29 CSCs spheroids were enriched according to our published protocol [38]. Briefly, detached single cells (0.05% trypsin/EDTA) were resuspended in DMEM/F12 (Serum-free medium, Gibco, Germany) supplemented with 20 ng/mL EGF (PeproTech, USA), 10 ng/mL bFGF (PeproTech, USA), 2% B27 complement (Gibco, Germany), and 1% NEAA. To prepare Hanging droplets of HT-29 cells, approximately 60 cellular drops (25 µL each) containing 5–10 × 10^3^ cells were placed over the lid of 10 cm Petri culture dishes prefilled with PBS, carefully turned upside down, and kept at 37 °C, 5% CO_2_, and a 95% humidified incubator for four days (96 h), then, transferred to low attachement plates using the supplemented DMEM/F12 medium [38].

#### Evaluation of potential CSCs markers

The expression of cancer stem cell surface markers in HT-29 spheroids was characterized using flow cytometry relative to the parental cell line. In brief, single cell suspensions of both spheroids and parental cell cultures were obtained and then subjected to two washes with cold phosphate-buffered saline (PBS) (Gibco, UK, #18912-014). Cell suspensions exhibiting over 95% viability, as verified by the trypan blue, were labeled to evaluate the expression of cancer stem cell markers using anti-DCLK1 (1:200), anti-CD166 (1:90), anti-CD133 (1:300), anti-CD44 (1:30), as primary antibodies (All obtained from Abcam, USA) for 30 to 60 min at 4 °C, followed by FITC-conjugated goat anti-rabbit IgG as the secondary antibody (1:100) (Santa Cruz Biotechnology, USA) for 30 min at 4 °C. In all experiments, non-immunized mouse or rabbit IgG was served as an isotype control instead of the primary antibody. The percentage of positive cells with the expression of CSCs markers was acquired utilizing an Attune NxT flow cytometer (Thermo Fisher Scientific, USA) and the data were subsequently processed by FlowJo VX software.

#### Cellular invasion assessment

The cell invasion test was carried out to evaluate the capacity of HT-29-derived spheroid cells to invade from the well inserts compared to the parental cell lines (HT-29 and Caco-2). Briefly, Matrigel was allowed to thaw at 4°C overnight and subsequently diluted with cold serum-free DMEM medium at a ratio of 1:4. Then 40 µL of a diluted solution of Matrigel was applied to the surface of the chamber of the 24-well transwell (12 inserts/24-well plate; 35224; SPL, Korea) and incubated at 37 °C for a duration of 6 h. The inserts were then placed in wells containing 400 µL of high-glucose DMEM medium containing 20% FBS. The detached cells (5- and 10 × 10^4^ cells) were washed and resuspended with 180 µL medium containing 1% FBS and placed at the uppermost part of the chambers covered with Matrigel. After 24 and 72 h of incubation, the transwell inserts were removed from the 24-well plate and subjected to two washing steps with PBS. Non-invaded cells were gently scraped from the superior part of the insert membrane by a cotton applicator soaked in a warm DMEM medium. The fixation of the invaded cells was achieved by immersing them in ice-cold methanol for 10 min, followed by two washing steps with PBS and staining with 0.5% crystal violet dye (0.05 g in 10 ml of methanol) for 10 min. After two washing steps, the inner membrane of the inserts was cut and placed on a slide and mounted. Finally, the number of invading cells was counted from images taken randomly from each group under a microscope and compared among the three cell groups. The MDA-MB-231 cell line, known for its high invasiveness in human breast cancer, was served as a positive control in all conducted invasion assays.

### A detailed methodological framework for isolating and characterizing exosomes

#### Isolating and purifying exosomes: an optimized protocol

Exosomes were isolated from cell supernatant and underwent purification as described previously [2]. Briefly, HT-29 and Caco-2 parental cell lines were cultured to approximately 70% confluence in DMEM/high glucose medium containing 10% FBS. After discarding the complete medium, the cells were washed three times with PBS and then treated with medium containing 10% exosome-depleted FBS (Gibco™, Germany) for 48 h. The supernatant was collected when the cells reached 90% confluence. To isolate exosomes derived from CSCs-enriched colonospheres, the serum-free supernatants of spheroids were collected and pooled during 10 days of culture. Harvested culture media from parental and spheroid cells were spun at 300 g for 10 min to remove cell debris, followed by concentration using a 100-kD MWCO Amicon Ultra capsule filter (Millipore, USA). Exosome purification was performed using the Exo-spin™ kit through precipitation according to the provided protocol (EXO1-8; Cell Guidance Systems, UK). Finally, the purified exosomes from each group were combined and stored at − 80 °C for subsequent use.

#### Exosome morphology assessment by scanning electron microscopy (SEM)

For assessing the morphological features of exosomes, glass slides were washed by immersing in 96% ethanol at − 20 °C overnight. The purified exosomes were incubated in 2.5% glutaraldehyde in PBS for 30 min and then dehydrated using a gradient of ethanol (50%, 70%, 80%, 90%, and 100%). All steps were carried out on a PBS-filled Petri dish to prevent rapid evaporation. The slides containing exosomes were sputter-coated with gold-palladium and observed under scanning electron microscopy (AIS-2100, SEM, Seron Technology, Korea).

#### Measuring size distribution: dynamic light scattering (DLS) methodology

To determine the size and diameter, the purified exosomes were diluted with PBS and subjected to DLS analysis on the Zetasizer Nano ZS (Malvern Instruments, Malvern, UK).

#### Western blot analysis of exosomal markers

To prepare cell lysate, harvested cells from parentals and CSCs_enr_-colonospheres were washed 3 times with PBS and subjected to ten freeze-thaw cycles using liquid nitrogen and a 37 °C water bath. After centrifugation at 20,000 g for 20 min, the supernatant was kept at − 80 °C. Exosomes were mixed with RIPA-5X lysis buffer (150 mM NaCl, 0.5% sodium deoxycholate, 1.0% Triton X-100, 0.1% SDS (sodium dodecyl sulfate), and 50 mM Tris, pH = 8.0) (Abcam, USA) and a protease inhibitor cocktail (Sigma, S8820, USA) and then centrifuged at high speed (> 12,000 g) for 10 min and the supernatant was collected. The protein concentration of isolated exosomes and cell lysates was measured using a bicinchoninic acid (BCA) protein assay kit (Takara, Japan). The resulting lysates were mixed with loading buffer and incubated for 5 min at 95 °C, then 15 µg of proteins from each sample were separated by 12% SDS-polyacrylamide gel electrophoresis. The electrophoresis gel was soaked in a blotting buffer for a few minutes, and proteins were transferred to a pre-wet polyvinylidene fluoride (PVDF) membrane at 100 V for 40–60 min at 4 °C. The membrane was stained with Ponceau S (CAS 6226-79; 114275, Merck, Germany) for 1 min, and strips of each sample were cut. After blocking with 5% skim milk for 2 h, PVDF was incubated with primary antibodies, including anti-CD63 and anti-CD81 (Exosomes Antibodies Array & ELISA Kits) (EXOAB-KIT-1, System Biosciences (SBI), UK, 1:1000) overnight, followed by incubation with secondary antibody (1:20000) (goat anti-rabbit IgG) for 1 h at room temperature (RT) (EXOAB-KIT-1, System Biosciences/SBI). Then, PVDF membranes were washed three times with PBS-Tween-20 and developed by enhanced chemiluminescence (ECL) substrate solutions (RPN3245; Amersham, UK) for 5 min, and chemiluminescence was detected using a LAS3000 instrument (Fujifilm, Japan). As a negative control, non-immune rabbit IgG was applied instead of the primary antibodies.

#### Exosome labeling and cell uptake assay

The exosome labeling was performed using lipid membrane fluorescent dye PKH26 (PKH26GL-Cell Linker Kit, Sigma-Aldrich/USA) according to the manufacturer’s instructions. Briefly, exosomes (20 µg) and 4 µL of PKH26 dye were separately diluted with 1000 µl of diluent. Exosome suspension and staining solution were combined and left to incubate for 1–5 min at RT. To stop the reaction, a matching volume of 1% BSA was added to the exosome suspension and left to incubate for 1 min, then transferred to 5 ml tubes, washed, and precipitated at 4 °C using ultracentrifugation at 100,000 g for 70 min (L8-70R Beckman/UltraCentrifuge) to remove any non-incorporated dye. The exosomes tagged with fluorescent dye were suspended in PBS (100 µL) and frozen at −80 °C for later use.

To track the exosome uptake by Caco-2 cells, 6 × 10^4^ cells were cultured in a nutrient-rich culture medium in 48-well culture plates and left to adhere for 18 h. The labeled exosomes (20 µg/mL) and PBS as control were added to the cell culture. To detect exosome uptake, cells were observed under a fluorescence microscope at 0, 2, 4, 8, and 24 h, followed by washing with warmed PBS when the maximum exosome uptake was observed. Next, cells were fixed with paraformaldehyde (4%) for 15–20 min at RT, and to stain the nuclei, cells were treated with 0.5 µg/mL of DAPI fluorescent dye (4’, 6-diamidino-2-phenylindole; Sigma-Aldrich/Germany) for 5 min at RT. The images were captured using a fluorescence-based microscope equipped with a 460 nm filter (Olympus IX71).

### Isolation and quantification of RNA: a detailed protocol for real-time quantitative PCR (qRT-PCR) analysis

#### Assessment of *KLF4*, *SOX2*, *c-MYC*, *NANOG* expression in HT-29 spheroid structures and HT-29 cell monolayer

To evaluate the stemness-associated gene expression—*KLF4*, *OCT4*, *SOX2*, *c-MYC*, and *NANOG*—in HT-29 spheroid structures compared to their parental cell lines, total RNA was extracted using Qiagen RNeasy Mini RNA Extraction System in accordance with the manufacturer’s guidelines. The quantity and quality of the extracted RNAs were evaluated using Thermo-Fisher Scientific Nanodrop and Electrophoresis on an agarose gel. Subsequently, DNase I digestion (1 U/µL) was performed to remove genomic DNA impurities, and cDNA synthesis (GeneAll, Korea) of 1 µg of total RNA was then carried out. To perform the real-time PCR amplification, the TaKaRa SYBR Premix Ex Taq II kit (Japan) and 10 mmol/L of each primer were applied and subjected to the PCR protocol with 40 cycles, each with a 5-second denaturation step at 95 °C and a 30-second annealing step at 60 °C on the Rotor-Gene Q Light Cycler (Qiagen, Germany). The primer sets used in this study are listed in Table 1S.

#### Quantification of ABC transporters expression *(B1*,* C1*,* G2*) in HT-29 spheroid cultures, HT-29, Caco-2 and exosomes-treated Caco-2 cell monolyers

To evaluate the expression of ABC efflux pump genes, namely *ABCB1*, *ABCC1*, and *ABCG2* in HT-29 spheroids, HT-29, Caco-2, and exosome-treated cells, total RNA was extracted, cDNA synthesized, and RT-qPCR conducted as described previously. Finally, the expression levels of the target genes were quantified using the 2−ΔΔCT method, normalized to glyceraldehyde-3-phosphate dehydrogenase (*GAPDH*) as the housekeeping gene. The primer sets used in this study are listed in Table 1S.

### Cell viability and MTT assay

#### Cell viability assay of exosome-treated cells

For evaluating the function of exosomes derived from different cell populations on the maintenance of viability of Caco-2 cells, the exosomes released from CSCs (HT-29-CSCs-EXOs), HT-29 parental cells (HT-29-EXOs), and Caco-2 parental cells (Caco-2-EXOs) were added to the cultured cells. In addition, Caco-2 cells were exposed to PBS as solvent and no-treatment controls (PBS-free). For this purpose, Caco-2 cell monolayers (5000 cells per well) were cultured in the 96-multiwell microplate for 18 h. Then, exosomes (10 µg/ml), as mentioned above, were added to the adherent cells. The viability of cells was measured at 24, 48, and 72 h by performing the MTT assay (3-(4,5-dimethylthiazol-2-yl)−2,5-diphenyltetrazolium bromide). Briefly, the supernatant was discarded and 100 µL of filtered MTT (5 mg/ml) was added to each well and incubated in 5% CO_2_ at 37 °C for 2–4 h. After discarding the MTT solution, dimethyl sulfoxide (DMSO) was added to each well on a shaker incubator for 15 min to dissolve formazan crystals. The absorbance readings were recorded at 570 and 630 nm using a microplate reader (1707203; Biotek/USA). The following formula was used to calculate cell viability: % of cell viability= (adjusted mean OD of sample—adjusted mean OD of solvent control) × 100 [39]. To confirm whether the observed effects were attributable to biologically active exosomal components, Caco-2 cells were treated with heat-inactivated exosomes (HI-EXOs) for 48 h, which were prepared by heating at 95 °C for 10 min prior to cell treatment.

#### Determination of drug half-maximal inhibitory concentration (IC50) in Caco-2 cells treated with Ebefluoro/5-FU (5-Fluorouracil)

To determine the 5-FU IC50 for Caco-2 cells, cells (5000 cells per well) were cultured in a 96-well plate and exposed to varying 5-FU concentrations (0, 1, 5, 10, 25, 50, 100, and 200 µg/mL) (A-4866; 50 mg/ml Ebefluoro/5-Fluorouracil; EBEWE Pharma, AUSTRIA) for 24, 48, and 72 h. The supernatant was then discarded and the MTT assay was performed.

#### Measurement of 5FU -IC50 in exosomes-treated Caco-2 cell monolayers

The Caco-2 cells were treated with 10 µg/mL of exosomes for 24 h followed by exposure to 10 µg/mL of 5-FU for 48 h, and then MTT assay was performed. The exosome dose of 10 µg/mL was selected based on the literature, where it has been the most frequently used dose to effectively induce biological responses in cancer cell lines, including CRC models, without cytotoxicity [40–42]. The treatment time of 48 h was chosen to allow sufficient exosome uptake and cellular response, consistent with previous studies [43–46].

The following experimental groups were considered: (1) Caco-2 cells + CSCs-EXOs + 5-FU; (2) Caco-2 cells + HT-29-EXOs + 5-FU; (3) Caco-2 cells + Caco-2-EXOs + 5-FU; (4) Caco-2 cells + PBS + 5-FU; (5) Caco-2 cells + 5-FU; (6) Caco-2 cells without any treatment; and DMEM with and without 5-FU were used as negative controls.

### Statistical analysis

Each experiment was conducted at least three times, and data were presented as mean ± standard deviation (SD). Paired Student’s t-tests were used for qRT-PCR and flow cytometry comparisons. One-way ANOVA with Bonferroni post hoc correction was applied to compare differences between multiple study groups. To represent multiple comparisons and reduce the risk of false-positive results (Type I error), p-values were adjusted using the Bonferroni correction. Non-linear regression and dose-response inhibition methods were used to calculate the IC50 of 5-FU. All statistical analyses were performed using GraphPad Prism version 8.0 (La Jolla, CA, USA). A p-value < 0.05 was considered statistically significant, with Bonferroni-adjusted p-values reported for multiple comparisons. For graphs, p-values < 0.05, < 0.01, < 0.001, and < 0.0001 were denoted by *, **, ***, and ****, respectively.

## Results

### Cell Culture, sphere formation and characterization of HT-29 spheroids as CSCs-like subpopulation

**Fig. 1:**
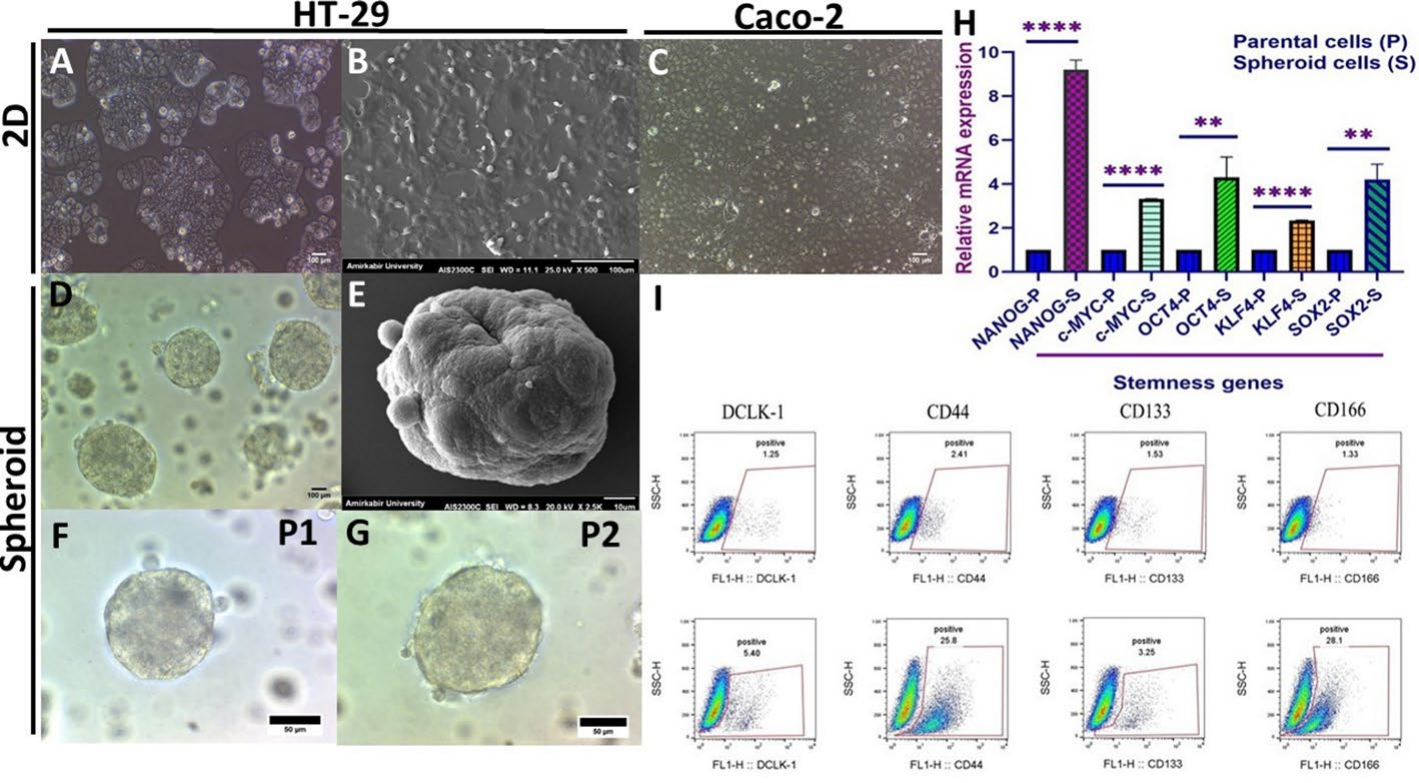
CSCs characteristics of HT-29 spheroid structures. 2D culture of colon adenocarcinoma cell lines; parental. **A** HT-29 spheroid structures with well-rounded and compact morphology. **B** SEM (Scanning Electron Microscope) imaging of HT-29 colon adenocarcinoma parental cells. **C** 2D culture of the Caco-2 colon adenocarcinoma parental cells. **D** HT-29 sphere formation assay showing well-rounded and compact morphology under light microscopy. **E** SEM (Scanning Electron Microscope) imaging method of derived-HT-29 spheroids. **F–G** Secondary HT-29 sphere formation assay (P1–P2 passages). **H** Real-time quantitative analysis of PCR exhibited a substantial increase in the relative expression of indicative stemness genes; *NANOG*, *c-MYC*, *OCT4*, *SOX2*, and *KLFA4* in derived-HT-29 spheroids (S) relative to their parental (P) counterparts normalized to *GAPDH*, presented as mean ± SD (*n* = 6). However, as noted, the level of KLF4 upregulation is lower compared to other stemness genes. **J** Flow cytometry plots of putative CRC-CSCs surface markers. Values under 0.0001 and 0.01 were denoted by the symbols ****, **, and ns as not significant, respectively

The HT-29 and Caco-2, two prominent and extensively utilized human colorectal cancer cell lines in CRC-related studies, were cultured under standard culture conditions (Fig. 1A, B). Cells cultured in routine two-dimensional culture were considered parental cells. To generate CSCs_−_enriched colonospheres from HT-29 cells, the hanging droplet and free-floating methods were applied consecutively in serum-free media containing EGF, bFGF, B27 supplement, and NEAA. The HT-29 cells formed compact and well-rounded spheroids (Fig. 1C, D). Analysis of expression levels of putative stemness genes revealed significantly higher expression of *NANOG* (*p-value* < 0.0001), *c-MYC* (*p-value* = 0.005), *OCT4* (*p-value <* 0.0001), *SOX2* (*p-value* = 0.007), and *KLF4* (*p-value* < 0.0001) in HT-29-derived spheroids relative to their parental monolayers. Although *KLF4* expression was relatively lower, it was significantly upregulated (*p* < 0.0001) in spheroid cells compared to parental cells after Bonferroni-correction analysis, supporting its role in CSCs enrichment alongside other stemness genes (*NANOG*, *OCT4*, *c-MYC*, and *SOX2*) (Fig. 1E).

**Fig. 2:**
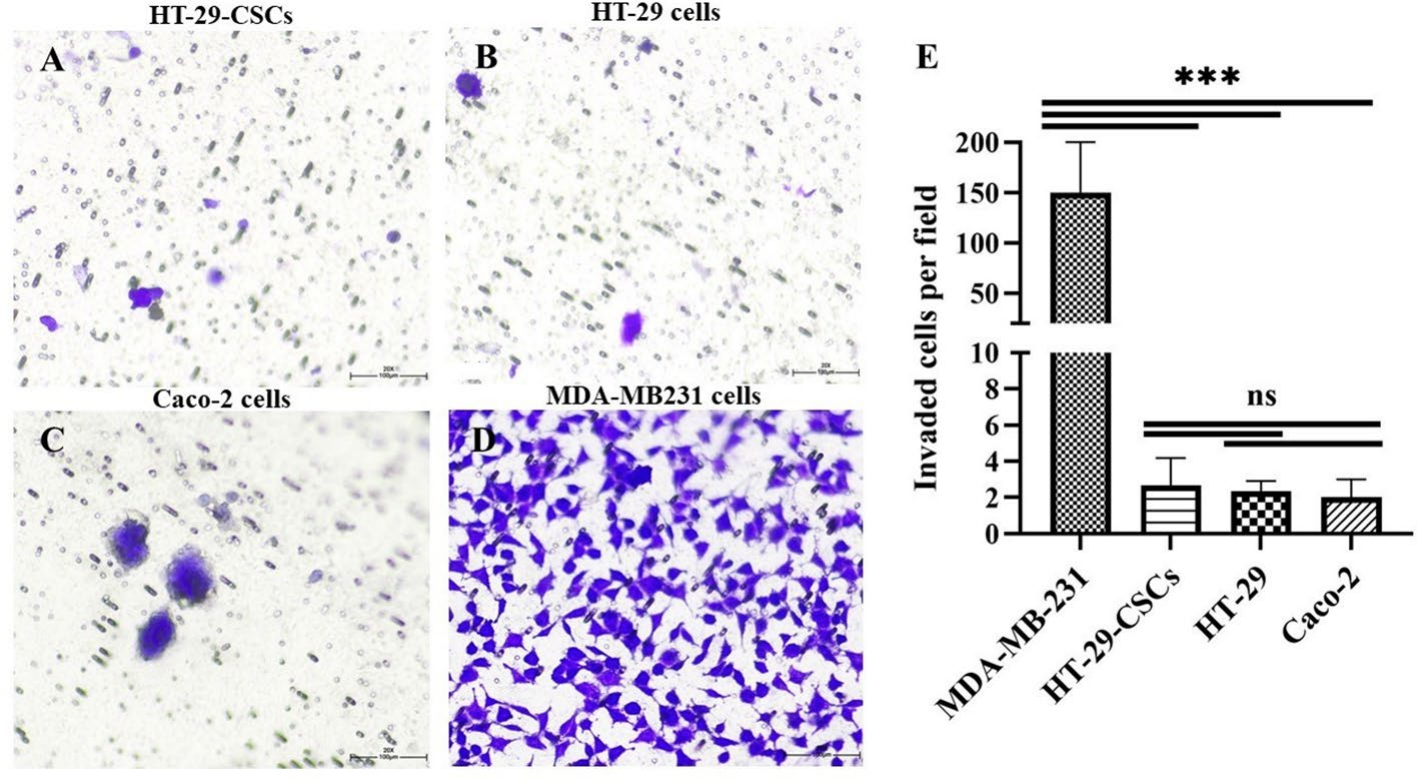
Invasion assay of parental HT-29 and Caco-2 monolayers and HT-29 spheroid structures. **A–D** Cell invasion assays using Matrigel-coated Transwell showed a significantly low number of invaded cells in HT-29-derived spheroid cells, parental HT-29 and Caco-2 monolayers. MDA-MB231 was selected as the positive control in the invasion assay (200X magnification). **E** No meaningful differences were detected across the three colorectal cell groups (*n* = 3)

To further validate the CSCs characteristics of the spheroids, the flow cytometry analysis showed a significant increase in the expression of potential CRC-CSCs (Fig. 1F), including CD166 (*p-value =* 0.003), CD44 (*p-value =* 0.001), CD33 (*p-value =* 0.006) and DCLK-1 (*p-value =* 0.011) in spheroid cells compared to their parental counterparts (Table 1). These results indicate that HT-29 spheroids exhibit enriched CSC properties as evidenced by the upregulation of stemness genes and increased expression of CSC surface markers. To evaluate the invasion potential of spheroid cells derived from HT-29 relative to HT-29 and Caco-2 monolayers, a Matrigel-coated transwell plate assay was performed. As depicted in Fig. 2A–E, the number of cells that invaded was low, with no substantial difference amongst the three groups (HT-29-CSCs, HT-29, and Caco-2), showing that HT-29-derived cell spheroids (HT-29-CSCs) do not exhibit enhanced invasiveness compared to parental cells. The positive control in this experiment was a breast cancer cell line of MDA-MB-231, which is known for its high invasiveness.

**Table 1:** The proportion of positive cells expressing CD44, CD166, CD133, and DCLK1 as common CRC-CSCs markers in HT-29 spheroid structures relative to their corresponding parental monolayers

Cells type	CSCs markers			
	CD44%**	CD133%^ns^	CD166%**	DCLK-1%*
Parental HT-29 cells	2.45 ± 0.06	1.265 ± 0.37	1.16 ± 0.23	1.27 ± 0.03
Spherical HT-29 cells	25.90 ± 0.14	3.37 ± 0.17	27.8 ± 0.42	5.50 ± 0.14

The data from theindependent experiments (*n* = 3) are outlined as mean ± SD%. Spheroids showed higher expression of the putative CRC-CSCs markers relative to their parental counterparts, (**p ≤ 0.01, *p ≤ 0.05, and ^ns^*p* > 0.05)

### Comparative expression assessment of *ABCB1*, *ABCC1* and *ABCG2* across experimental groups of HT-29 spheroid structurs, HT-29 and Caco-2 monolayer cells

**Fig. 3:**
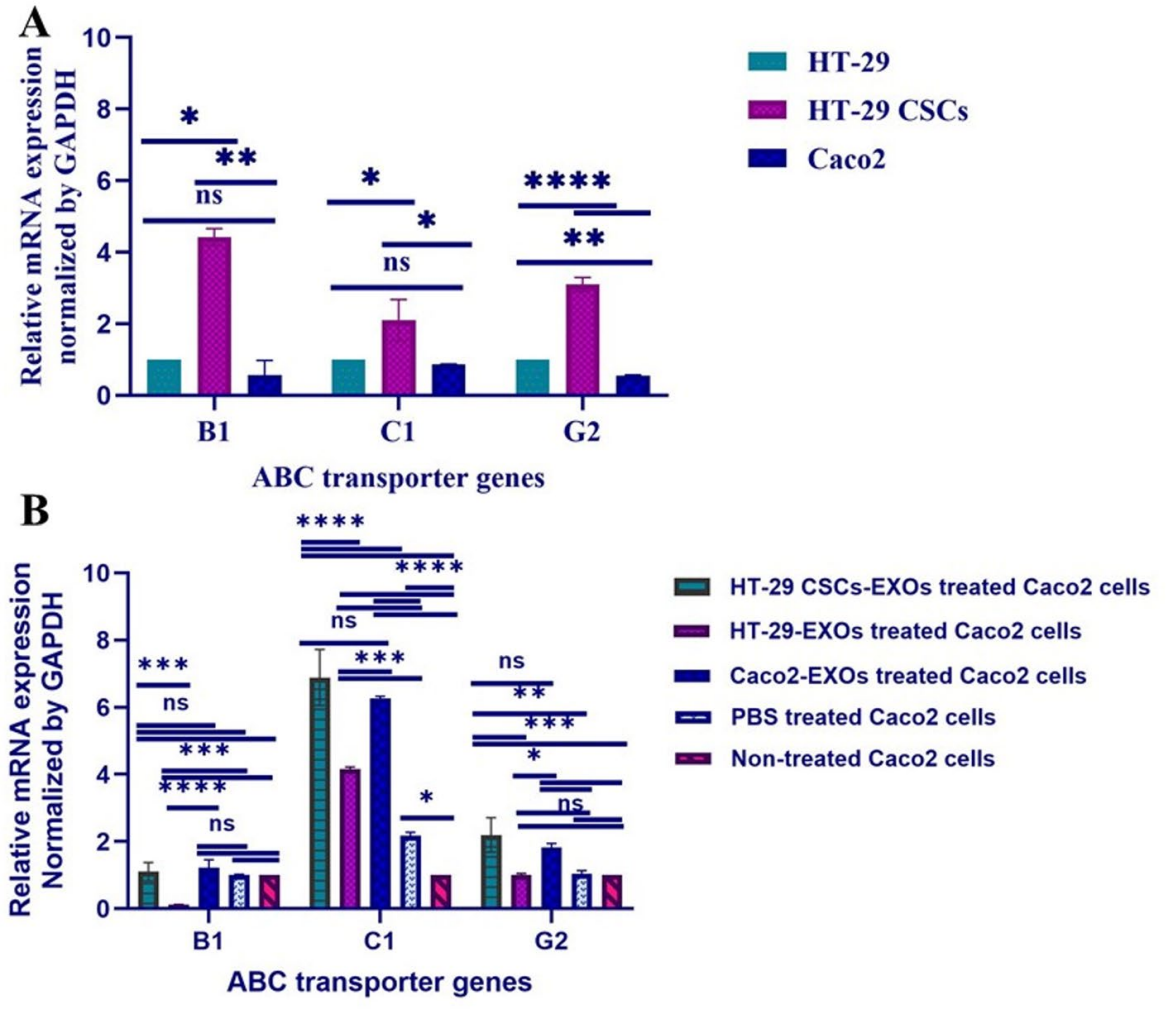
The level of expression of ABC drug resistance transporters in HT-29 CSCs-enriched spheroid structures relative to their adherent counterparts (HT-29 and Caco-2) and in Caco-2 monolayers treated with exosomes. **A** Real-time quantitative PCR demonstrated a substantial increase in the relative expression of transporter genes; *ABCB1*, *C1* and *G2* in HT-29 CSCs-enriched spheroids relative to their parental monolayers, normalized to *GAPDH*, presented as mean ± SD (*n* = 2). **B** The level of *ABCC1* expression in Caco-2 cells treated with exosomes derived from HT-29 CSCs (HT-29 CSCs-EXOs) and exosomes derived from Caco-2 cells (Caco2-EXOs) was significantly increased compared to control groups. A marked difference was detected in the levels of *ABCC1* and *ABCG2* gene expression in Caco-2 cells treated with HT-29 CSCs-EXOs compared to non-treated cells. Exosomes derived from adherent HT-29 cells (HT-29 parental-EXOs) were used as an additional comparison group. The p-values less than 0.05, 0.01, 0.001, and 0.0001 are indicated on the graph using *, **, ***, and ****, respectively, and ns = not significant (*n* = 3)

The expression of the ABC transporters; *B1*, *C1*, and *G2* as drug resistance genes were significantly higher in HT-29 CSCs-enriched spheroids compared to HT-29 (*p-value* = 0.0286, 0.0156, and < 0.0001, respectively) and Caco-2 (*p-value* = 0.0040, 0.0190, and < 0.0001, respectively) parental cells. In addition, the gene expression levels of *ABCG2* varied significantly (*p-value* = 0.0063) between HT-29 and Caco-2 monolayer cells. Whereas, the increase in expression levels of *ABCB1* (*p-value* = 0.206) and *ABCC1* (*p-value* = 0.869) was not statistically significant between HT-29 and Caco-2 monolayer cells (Fig. 3A). This suggests that HT-29 spheroids not only possess stem cells-like traits but also a heightened drug resistance profile, which is a characteristic of cancer stem cells. Furthermore, treatment of Caco-2 monolayer cells with exosomes derived from HT-29 CSCs (HT-29 CSCs-EXOs) significantly increased ABCC1 expression compared to untreated cells and Caco-2 cells treated with Caco-2-derived exosomes (Caco2-EXOs). ABCG2 expression was also elevated in Caco-2 cells treated with HT-29 CSCs-EXOs, whereas exosomes from parental HT-29 cells induced a smaller effect. These results indicate that CSC-derived exosomes can modulate ABC transporter expression in recipient Caco-2 cells (Fig. 3B).

### Characterization of exosomes released by CRC cell lines and CSCs enriched colonospheres

**Fig. 4:**
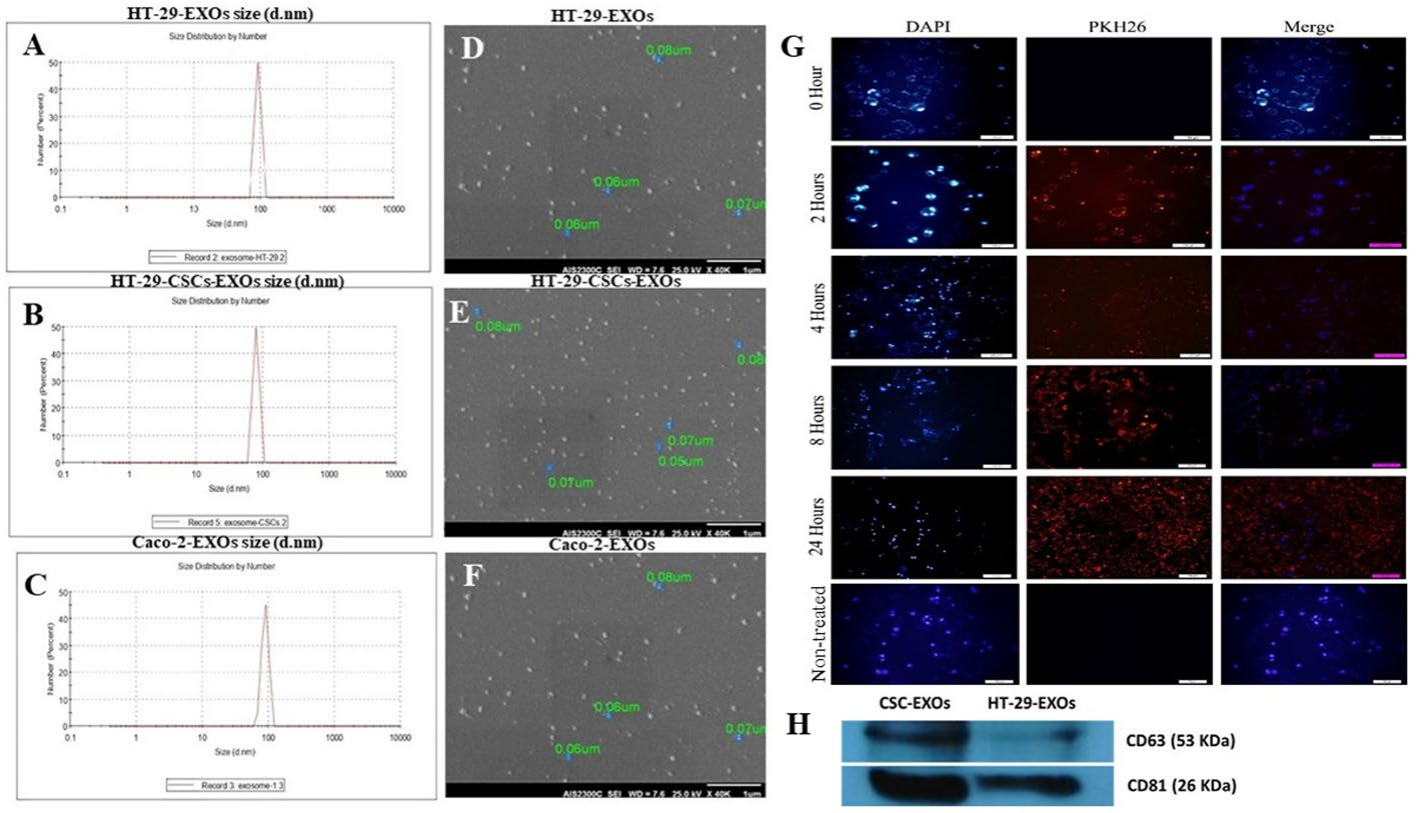
Characterization of purified exosomes from CRC cell lines (HT-29 and Caco-2) and HT-29-derived spheroids. **A–C** Size distribution of exosomes from HT-29 cells (HT-29-EXOs), spheroids (CSCs-EXOs), and Caco-2 cells (Caco-2-EXOs) measured by dynamic light scattering (91.93 ± 9.65, 79.44 ± 8.38, and 88.28 ± 10.76 nm, respectively). **D–F** Scanning electron micrographs of the exosomes from HT-29-EXOs, CSCs-EXOs, and Caco-2-EXOs (scale bar = 1 μm), respectively. **G** Uptake of PKH26-labeled exosomes by Caco-2 cells at 0, 2, 4, 8, and 24 h; non-treated cells served as negative control. **H** Western blot analysis of exosomal markers (CD63/CD81). Adapted from: Naseri et al. [2]. ©2021 The Authors. Published by John Wiley & Sons Ltd and distributed under the terms of the Creative Commons Attribution License (CC BY)

To compare the effect of exosomes released by parental (HT-29 and Caco-2) and spheroid cells on the survival and drug resistance of Caco-2 cell monolayers, exosomes were isolated from the culture supernatants of all three cell groups, and the characteristics of isolated exosomes were assessed. Analysis of DLS data showed range of sizes of 91.93 ± 9.65 nm and 79.44 ± 8.38 nm diameter for HT-29-EXOs and CSCs-EXOs, respectively (Fig. 4A, B). The derived exosomes from Caco-2 cells (Caco-2-EXOs) revealed a size range of 88.28 ± 10.76 nm in diameter (Fig. 4C). Electron microscopy demonstrated that all purified exosomes had typical round morphology with sizes similar to those obtained by DLS (Fig. 4D–F). The mean size of CSCs-EXOs was significantly smaller than that of HT-29-EXOs and Caco-2-EXOs, (*p-value ≤* 0.0001) (Fig. 1S).

The fluorescent microscopy images showed that approximately 70% of Caco-2 cells efficiently internalized the PKH26-labeled exosomes after 8 h of exposure. As shown in Fig. 4G, the maximum uptake was recorded 24 h after exposure of Caco-2 cells to the labeled exosomes, indicating that Caco-2 cells internalize cancer-derived exosomes. The exosomal markers, CD81 and CD63, were detected in CSCs-EXOs and parental-EXOs using western blot analysis (Fig. 4H), as previously reported [2]. These results confirm the proper isolation of exosomes with characteristic morphology, size, marker expression, and successful internalization by recipient cells.

### Exosome treatment and its impact on drug resistance genes: expression alterations in Caco-2 cells

Based on the results regarding the higher expression of drug resistance genes in spheroid cells as a cancer stem cell-like subpopulation compared to non-cancerous stem cells, the question was raised whether this feature could be transferred by exosomes to non-cancer stem cells. Therefore, we assessed the expression of Genes encoding ABC Transporters: *ABCB1*, *ABCC1* and *ABCG2* in Caco-2 cells treated with HT-29-CSCs-EXOs, HT-29-EXOs and Caco-2-EXOs. As shown in Fig. 3B, higher gene expression levels of *ABCB1*, *ABCC1*, and *ABCG2* were observed in Caco-2 cells treated with CSCs-EXOs compared to those treated with parental HT-29-EXOs (*p-value* = 0.0002, ≤ 0.0001, 0.0009), Caco2-EXOs (*p-value* = 0.8573, 0.3264, 0.3876), PBS (*p-value* = 0.9489, ≤ 0.0001, 0.001), and non-treated cells (*p-value* = 0.927, ≤ 0.0001, 0.0008) (Fig. 3B). In addition, the expression levels of *ABCB1*, *ABCC1*, and *ABCG2* were significantly higher in Caco-2 cells exposed to Caco2-EXOs relative to the HT-29-EXOs treated cells (*p-value* ≤ 0.0001, 0.0003, 0.013), PBS (*p-value* = 0.4778, ≤ 0.0001, 0.016), and non-treated cells (*p-value* = 0.437, ≤ 0.0001, 0.011). Therefore, exosomes derived from CSC-enriched spheroids may contain a drug resistance-related gene expression profile compared to parental cancer cells.

### Effect of exosome treatment on proliferation of Caco-2 cell monolayers

**Fig. 5:**
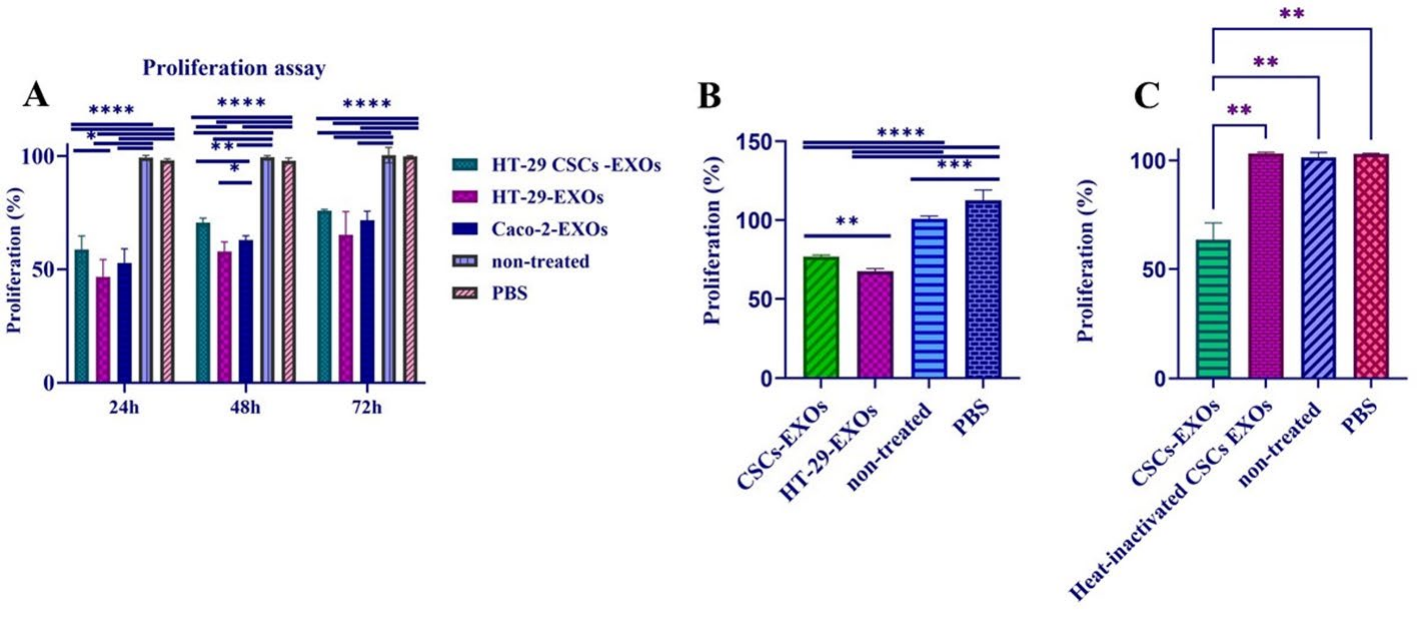
CSCs-, HT-29- and Caco-2-derived exosomes decreased the proliferation of Caco-2 and HT-29 cells. **A** Comparative analysis of Caco-2 cell proliferation after treatment with exosomes at different time intervals (24, 48, and 72 h) (*n* = 3). As observed in the graph, exosome-treated groups (CSCs-, HT-29-, and Caco-2-derived exosomes) showed decreased cell proliferation compared to untreated groups and PBS. Cell proliferation was higher in cells treated with CSCs- derived exosomes compared to cells treated with HT-29-, and Caco-2-derived exosomes. **B** Comparative analysis of HT-29 cell proliferation after treatment with exosomes at 48 h (*n* = 3). Exosome-treated groups showed a significant decrease in cell proliferation compared to untreated and PBS-treated groups. Following Bonferroni correction, the difference in cell proliferation was statistically significant (*p* < 0.05) between CSCs-EXOs and HT-29-EXOs treated groups. The p-values less than 0.05, 0.01, 0.001, and 0.0001 are shown using *, **, ***, and ****, respectively, on the graphs (*n* = 3). **C** Comparative analysis of cell proliferation following 48 h treatment with CSC-derived exosomes (CSCs-EXOs), heat-inactivated exosomes (HI-EXOs; 95 °C for 10 min), or PBS as a control. Significant reductions in proliferation were observed in cells treated with CSC-EXOs, whereas HI-EXOs failed to induce this effect and showed proliferation levels comparable to the control (p-value < 0.01). These results indicate that the antiproliferative response depends on the biological activity and functional integrity of exosomal biomolecules. Data represent mean ± SD from three independent experiments; *p* < 0.05 versus the control

The monolayer cells of HT-29 and Caco-2 were treated with 10 µg/ml of exosomes and subsequent experiments were performed at 24, 48, and 72 h after the addition of the exosomes. To explore the impact of exosome uptake on cell proliferation, the appropriate number of Caco-2 cells (5000 cells per well) were cultured and cells were exposed to exosomes for 24, 48, and 72 h according to the mentioned groups. It should be noted that the MTT assay reflects cell viability based on metabolic activity, which is an indirect measure of cell proliferation. Therefore, treatment-induced metabolic alterations may partially contribute to the exosome-mediated effects. Cell proliferation of Caco-2 cells treated with CSCs-EXOs and parental HT-29-EXOs, and Caco-2-EXOs in all three incubation time points was considerably decreased relative to the control group (*p-value <* 0.0001, *p-value =* 0.0006, *p-value =* 0.003) and PBS (*p-value <* 0.0001, *p-value =* 0.001, *p-value =* 0.004), respectively (Fig. 5A). The cells treated with HT-29-EXOs and Caco-2-EXOs showed a further decrease in proliferation, which was statistically significant compared to the cells treated with CSCs-EXOs. The exosomal uptake did not affect the cellular phenotype of the recipient cells (data not shown). To analyze the effects of exosomes on cell proliferation more thoroughly, HT-29 cells were treated with CSCs-EXOs and HT-29-EXOs. As shown in Fig. 5B, cells treated with CSCs-EXOs and HT-29-EXOs exhibited a substantial decrease in cell proliferation relative to PBS (*p-value <* 0.0001 and *p-value =* 0.018) and non-treated control (*p-value =* 0.001 and *p-value =* 0.002), respectively. After Bonferroni correction, the difference in cell proliferation between CSCs-EXOs and HT-29-EXOs treated groups was statistically significant (*p* < 0.05). To validate whether the observed effects were due to biologically active exosomal components, CSC-derived exosomes were heat-inactivated at 95 °C for 10 min prior to cell treatment. As shown in Fig. 5C, after 48 h treatment, the proliferation rate of cells exposed to heat-inactivated exosomes (HI-EXOs) was nearly identical to that of the PBS-treated and non-treated controls. In contrast, treatment with intact CSCs-EXOs resulted in a significant reduction in proliferation (p-value < 0.01).

### Effect of CSCs-, HT-29- and Caco-2 exosomes on 5-Fu treated Caco-2 cell monolayers

**Fig. 6:**
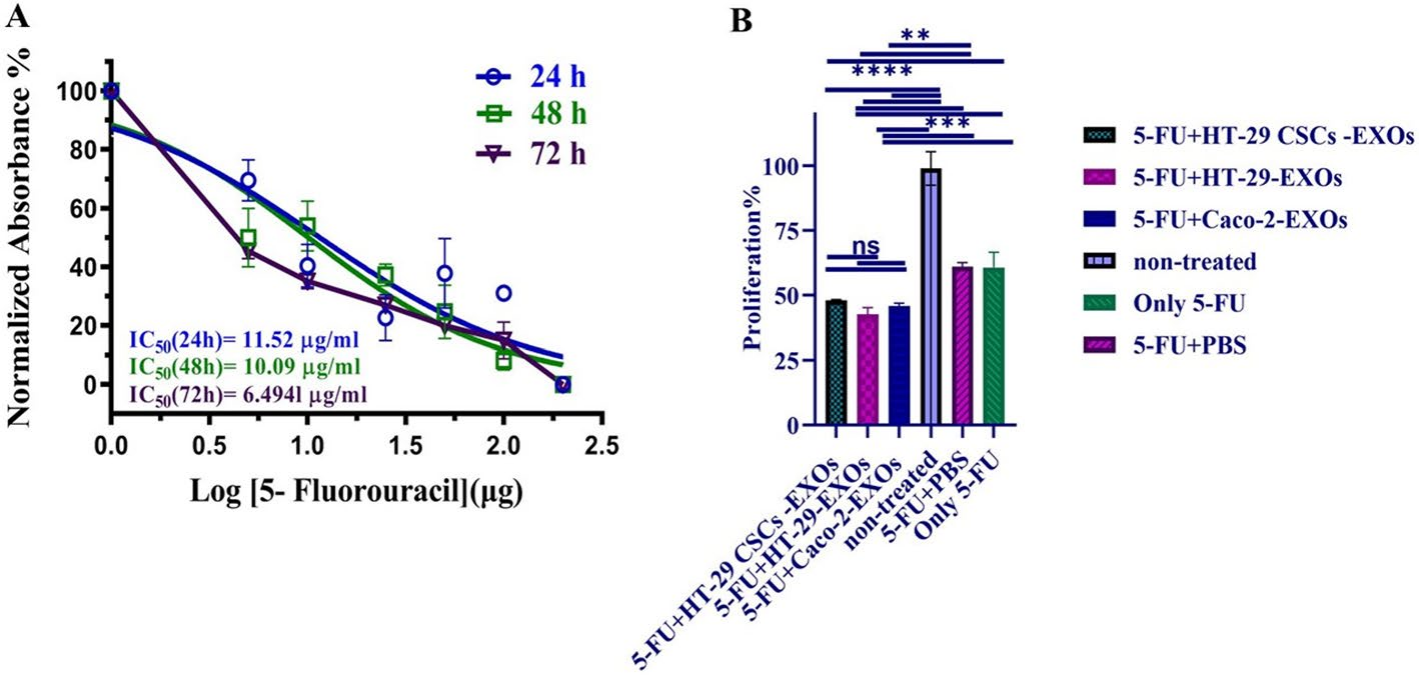
The cellular viability of Caco-2 cells treated with 5-FU chemotherapy drug and the effect of exosomes on drug cytotoxicity properties of Caco-2 cells. **A** IC50 and R2 square for different time points of 5-FU (24, 48, and 72 h) were 11.52 (R2 = 0.8148), 10.09 (R2 = 0.9020), and 6.4941 (R2 = 0.9130), respectively. Cellular viability was quantified by MTT assay. Data were recorded as the mean ± SD. **B** Comparative analysis showed that Caco-2 cell proliferation was significantly reduced after 5-FU treatment in exosome-treated groups (CSCs-, Caco-2- and HT-29-EXOs) compared to the control groups. While, cell proliferation was higher in cells treated with 5-FU + H29 CSCs-EXOs compared to cells treated with 5-FU + Caco-2-Exos and 5-FU + Caco-2-EXOs, but it was not statistically significant. *P-values* less than 0.05, 0.01, 0.001 and 0.0001 were shown using *, **, ***and ****, respectively, on the graphs (*n* = 3)

To explore whether exosome treatment could modulate the sensitivity of Caco-2 cells to the chemotherapy drug 5-FU, the cytotoxicity of 5-FU in non-treated Caco-2 cell monolayers was first determined. The cytotoxicity of 5-FU in Caco-2 cells was evaluated based on its effect on cell growth using the MTT assay. According to Fig. 6A, the half-maximal inhibitory (IC50) concentration of 5-FU on Caco-2 cells was 11.52, 10.09, and 6.49 µg/ml after 24, 48, and 72 h of incubation, respectively. These findings show a baseline cytotoxic response of Caco-2 cells to 5-FU and show increasing cytotoxicity with longer exposure. Afterward, Caco-2 cells were treated with different exosome preparations (CSCs_−_enriched colonospheres, parental cells of HT-29, and Caco-2 cells) and 5-FU.

Our results revealed that the Caco-2 cell monolayers treated with exosomes from CSCs_−_enriched colonospheres, HT-29 and Caco-2 cell monolayers showed lower viability compared to the non-exosome-treated group (*p-value <* 0.0001) (Fig. 6B). Notably, CSCs-EXOs showed a lesser reduction in Caco-2 cell viability compared to the groups treated with HT-29-EXOs and Caco-2-EXOs; however, this difference was not statistically significant. Similarly, the Caco-2 cells treated with only 5-FU showed higher viability than Caco-2 cells treated with exosomes obtained from CSCs_−_enriched colonospheres (*p-value* = 0.0014), HT-29 (*p-value <* 0.0001), and Caco-2 cells (*p-value* = 0.0003). Following exosome treatment, an increase in the expression of drug resistance genes was observed. However, the MTT assay (cell viability) showed a decreased signal in cells treated with exosomes followed by 5-FU, as shown in Fig. 6B.

## Discussion

Tumor growth and development arise from intricate communications between tumor cells, CSCs, and tumor microenvironment [47]. These dynamic interactions can alter cell phenotype or function continuously [48]. Exosomes serve a central role in cell-to-cell communication locally and systematically [49]. Some lines of evidence have revealed that the derived exosomes from cancer cells involve in neoplastic progression, organotropic metastasis [50], angiogenesis [51], dormancy [52], escape from the immune system [53], migration, programmed cell death, apoptosis, drug resistance, plasticity, and invasion [18]. Extensive studies have shown that CSCs contribute to tumor recurrence, dissemination, and metastasis [54]. However, few studies have addressed the role of CSCs-derived exosomes on tumor cell behavior, and there are still unknown aspects regarding CSCs-exosomes and their potential involvement in cancer development [3, 16, 55]. Our previous study indicated that the levels of expression of drug resistance genes, namely ABC transporters, were significantly higher in HT-29-CSCs-enriched colonospheres than in their parental HT-29 monolayer cells [38]. Therefore, this research was conducted to explore the potential of CSCs exosomes in inducing drug-resistant features in a different colorectal cancer cell line.

As the first steps, CSCs-enriched spheroids were developed from the HT-29 cell line using the low-attachment method and serum-free conditions as a commonly used sphere culture system. The application of the spheroid model for the isolation of CSCs offers a significant advantage compared to relying only on CSCs surface markers. It enables the enrichment of whole subpopulations of CSCs that are more representative of in vivo tumor characteristics [56]. We observed a significant increase in KLF4 expression in HT-29-derived spheroids compared to parental cells after applying Bonferroni correction (*p* < 0.0001; Fig. 1E), which supports its potential role in CSC enrichment alongside other stemness genes such as *NANOG*, *OCT4*, *c-MYC*, and *SOX2*. However, *KLF4* upregulation was lower relative to these genes, reflecting the known heterogeneity of *KLF4* expression within CSC populations, as reported in recent studies [57, 58].

Considering the importance of CSCs in cell invasion, we are inclined to assess the invasion ability of spheroids cultured from HT-29 cell monolayers alongside the parental cells (HT-29 and Caco-2). The invasion assay revealed no significant difference in invasive capacity between the HT-29-derived spheroids and the parental HT-29 cells (Fig. 2). While invasion is commonly associated with CSCs, our results suggest that the CSCs population in HT-29 spheroids lacks an invasive phenotype. Our data highlight other prominent CSC traits, including self-renewal and drug resistance gene expression.

To isolate exosomes, we used the Exo-spin™ kit, which combines precipitation and is considered one of the rapid and most convenient methods for a range of research applications [59]. Aligning with previous reports, the isolated CRC exosomes were validated by common methods, including TEM, DLS, and western blotting to confirm their proper purification [2, 18]. The size distributions of the exosomes released by HT-29, Caco-2, and spherical clusters were consistent with the expected characteristics of exosomes, with a significant difference in mean size of CSCs-EXOs compared to HT-29- and Caco-2-EXOs. CSC-derived exosomes (CSC-EXOs) upregulated the expression of *ABCB1*, *ABCC1*, and *ABCG2* in Caco-2 cells relative to those exposed to their parental-EXOs, suggesting a role in promoting drug resistance. Although identifying the specific molecular mediators was beyond the scope of this work, previous studies have shown that exosomal microRNAs—such as miR-1246, miR-19b-3p, and miR-21—can modulate ABC transporter expression by targeting negative regulators or activating Wnt/β-catenin and PI3K/AKT pathways [60, 61]. Similarly, exosomal lncRNAs such as H19, UCA1, and AGAP2-AS1 have been shown to enhance chemoresistance by modulating ABC transporter expression and function [62, 63]. These findings underscore the importance of exosome-mediated RNA transfer in the regulation of multidrug resistance [21]. Interestingly, we observed an increased expression of drug resistance gene regulation in Caco-2 cells exposed to Caco-2 exosomes. This may be due to the increased uptake of Caco-2-EXOs by Caco-2 monolayer cells. Some lines of evidence indicated that exosomes originating from a specific cell type are more likely to be taken up by the same cell type [64]. Furthermore, we noted a reduction in cellular proliferation in Caco-2 cells exposed to HT-29 CSCs-EXOs and their parental EXOs relative to the non-treated control group. Since the MTT assay reflects metabolic activity rather than direct proliferation, part of the observed reduction in MTT signal may not exclusively reflect decreased cell proliferation but may also result from treatment-induced alterations. Future work will incorporate complementary proliferation-specific assays to further validate these findings. However, this decrease was less pronounced in the cells treated with HT-29 CSCs-EXOs. While CSC-derived exosomes are commonly linked to tumor progression, their cargo may also induce regulatory effects such as growth arrest, depending on the recipient cell type and internal signaling status [65, 66]. Exosomes have been shown to influence cellular processes through several pathways and molecules, including cell proliferation and cell cycle arrest in esophageal carcinoma (EC) cells [18]. Therefore, a similar phenomenon may have occurred in our experiment as well, although we did not evaluate it. Future studies incorporating cell cycle and apoptosis assays are warranted to elucidate the specific pathways involved. To further confirm the specificity of the exosomes mediated effects, an additional experiment was performed using heat-inactivated CSC-derived exosomes. Unlike cells treated with intact CSC-EXOs, cells exposed to heat-inactivated exosomes showed proliferation levels similar to controls. The loss of the anti-proliferative effect, accompanied by an increased MTT signal in cells treated with heat-inactivated CSCs exosomes, further supports the idea that observed biological activity depends on the functional integrity of exosomal biomolecules.

Interestingly, co-treatment with CSC-derived exosomes and 5-FU resulted in a greater reduction in viability than treatment with 5-FU alone. In this study, despite the observed upregulation of drug resistance-related genes in colorectal cancer cells treated with exosomes, a significant decrease in signal was detected by the MTT assay. This phenomenon may be attributed to the induction of cell cycle arrest by the exosomal contents [61, 67–69], rendering a substantial fraction of cancer cells quiescent and less susceptible to 5-FU, which primarily targets rapidly dividing cells [70, 71]. Therefore, the reduced cell viability observed in the MTT assay likely reflects suppressed proliferation rather than a decrease in drug resistance [46, 72–74]. Furthermore, increased mRNA expression of drug resistance genes does not necessarily translate into immediate functional protein activity due to post-transcriptional and post-translational regulatory mechanisms [75]. These findings highlight a complex cellular adaptation to exosome-mediated signaling, thereby providing a nuanced understanding of the role of exosomes in drug resistance and tumor cell proliferation. To this point, few studies have examined the impact of CSCs-EXOs or CSCs-microvesicles on phenotypical and functional changes in various tumor cells, with a focus on kidney [19], prostate [36], renal cell carcinoma [16], and thyroid [37]. However, cell proliferation and alternation in drug resistance gene have not been assessed. The current study presents a novel viewpoint on the function of CSCs-EXOs in altering cell phenotype and function. Recent studies suggest that the biological effects of exosomes on recipient cells are not always the result of a simple transfer of functional mRNA or proteins. In many cases, exosomal cargo contains regulatory molecules, such as miRNAs or signaling modulators, that can influence gene expression indirectly [42, 76–78]. For example, it has been reported that exosomal miR-21 promotes stem-like traits and chemoresistance in cancer cells by activating Wnt/β-catenin signaling, despite not containing stemness markers itself [77]. These findings underscore the importance of characterizing the molecular content of CSC-derived exosomes, including stemness-related proteins and RNAs, to better understand their functional impact on recipient cells. 

**Limitations of the study**

While our findings suggest a potential role of CRC CSC-derived exosomes in drug resistance and reduced proliferation, several limitations must be acknowledged [1]. The evaluations of drug resistance-related genes in this study were limited to mRNA expression analysis using qRT-PCR. While this method provides insight into transcriptional regulation, it may not directly reflect changes at the protein or functional level. Although we utilized the MTT assay to evaluate cell viability, this method alone cannot fully capture the complexity of drug response mechanisms. Future studies should incorporate complementary approaches such as clonogenic survival assays and western blotting, to analyze changes at protein level associated with survival and drug resistance pathways [2]. The present study did not include cell cycle analysis; however, incorporating both cell cycle and apoptosis assays in future investigations would help clarify the underlying mechanisms and strengthen the biological interpretation of the findings [3]. While Exo-spin™ provides a practical and reproducible approach, ultracentrifugation remains the gold standard for exosome isolation [4]. Comprehensive EV characterization, including assessment of markers such as TSG101, ALIX, Calnexin, and GM130, was not performed [5]. Since the cargo of exosomes—including proteins, RNAs, and other bioactive molecules—can strongly influence recipient cell behavior, a detailed characterization and comparison of exosomal content is essential for identifying the specific mediators responsible for the exosome-mediated effects [6]. Although our results suggest a paradoxical role of CSC-derived exosomes in both promoting resistance and reducing proliferation, further validation in in vivo models and across multiple CRC cell lines is necessary to generalize these findings and assess their translational relevance.

## Conclusion

In conclusion, CSCs-derived exosomes can alter the gene expression profiles and phenotypes of tumor cells, thereby affecting tumor development. Our findings indicate that exosomes play a multifaceted role in modulating drug resistance and cell proliferation in colorectal cancer cells. Although exosome-treated cells exhibited upregulation of drug resistance-related genes, MTT assay results revealed a significant decrease in cell viability following 5-FU treatment compared to controls. This apparent contradiction may be explained by exosome-mediated effects, which renders a substantial fraction of cells less responsive to 5-FU, a drug that targets actively dividing cells. Thus, the reduced cell viability likely reflects suppressed proliferation rather than effective drug resistance, despite the observed changes in gene expression. Additionally, the increased mRNA levels of resistance genes may not translate immediately into functional protein expression due to various post-transcriptional and translational regulatory mechanisms. Notably, exosomes derived from CSCs showed a slightly greater inhibitory effect on cell viability compared to those from parental cells, suggesting a more pronounced biological impact. Collectively, these findings underscore the multifaceted nature of exosome-mediated signaling and suggest a potential mechanism of tumor cell phenotype switching via transfer of specific molecular cargo, particularly from CSC-derived exosomes. Future studies should prioritize investigating the molecular dynamics of CSCs and tumor cells plasticity mediated by exosomes.

## Supplementary Information

Below is the link to the electronic supplementary material.

Supplementary Material 1

## Data Availability

The analyzed data during the current study are available from the corresponding author on reasonable request.

## Abbreviations

CSCs: Cancer stem cells
CRC: Colorectal cancer
CSCs-EXOs: CSCs-derived exosomes
CSCs-EXOs: Exosomes released by CSCs
HT-29- and Caco-2-EXOs: HT-29 CSCs-EXOs and parental cell exosomes
DLS: Light dynamic scattering
5-FU: 5-Fluorouracil/Ebefluoro
EMT: Epithelial to mesenchymal transition
TEXs: Tumor-derived exosomes
EVs: Extracellular vesicles
non-GSCs: Non glioblastoma stem cells
ESCC: Esophageal squamous-cell carcinoma
CCRCC: Clear-cell renal cell carcinoma
IBRC: Iranian Biological Resource Center
DMEM: Dulbecco’s modified Eagle’s medium
PBS: Phosphate buffer saline
MWCO: Molecular weight cut-off
SEM: Scanning electron microscopy
SDS: Sodium-dodecyl-sulfate
BCA: Bicinchoninic acid
PVDF: Pre-wet poly-vinyl-idene fluoride
RT: Room temperature
ECL: Enhanced chemiluminescence
PKH26 Red Fluorescent Cell Linker Kits: Lipid membrane dye PKH26
DAPI: 4′,6-diamidino-2-phenylindole
MTT: 3-(4,5-dimethylthiazol-2-yl)-2,5-diphenyltetrazolium bromide
DMSO: Dimethyl sulfoxide
IC50: Half-maximal inhibitory concentration
qRT-PCR: Quantitative Real‑Time PCR analysis
GAPDH: Glyceraldehyde-3 phosphate dehydrogenase
SD: Standard deviation
2D: Two-dimensional
3D: Three-dimensional
S: Spheroids
P: Parental
MSCs: Mesenchymal stromal cells
EC: Esophageal carcinoma
ECRG4: EC-related gene 4
BC: Breast cancer
hNSCs: Human neural stem cells
hNSC-EXOs: Human neural stem cell exosomes
ABC transporters: Genes related to drug resistance
ABC: ATP-binding cassette
ABCB1: ATP-Binding Cassette Subfamily-B Member 1
ABCC1: ATP Binding Cassette Subfamily C Member 1
ABCG2: ATP Binding Cassette Subfamily G Member 2
C-MYC: C-MYC proto-oncogene
E-cadherin: Epithelial Cadherin
FBS: Fetal bovine serum
KLF4: Kruppel-Like Factor 4
NANOG: Nanog homeobox
N-cadherin: Neural Cadherin
OCT-4: Octamer-binding transcription factor 4
SNAIL1: Snail family zinc finger 1
SOX2: SRY-Box Transcription Factor 2
TWIST1: Twist family bHLH transcription factor 1
ZEB1: Zinc finger E-box homeodomain 1
poly-HEMA: Poly-2-Hydroxyethyl Methacrylate
EGF: Epidermal Growth Factor
bFGF: Basic Fibroblast Growth Factor
FDA: Food and Drug Administration
