# Supplementary figures and images for: Functional role of cancer stem cell like exosomes on survival and drug resistance behaviors of colorectal cancer cells
Source: Discov Oncol. 2025 Dec 20;17:180. doi: 10.1007/s12672-025-04295-0 (PMC12855717; doi:10.1007/s12672-025-04295-0)

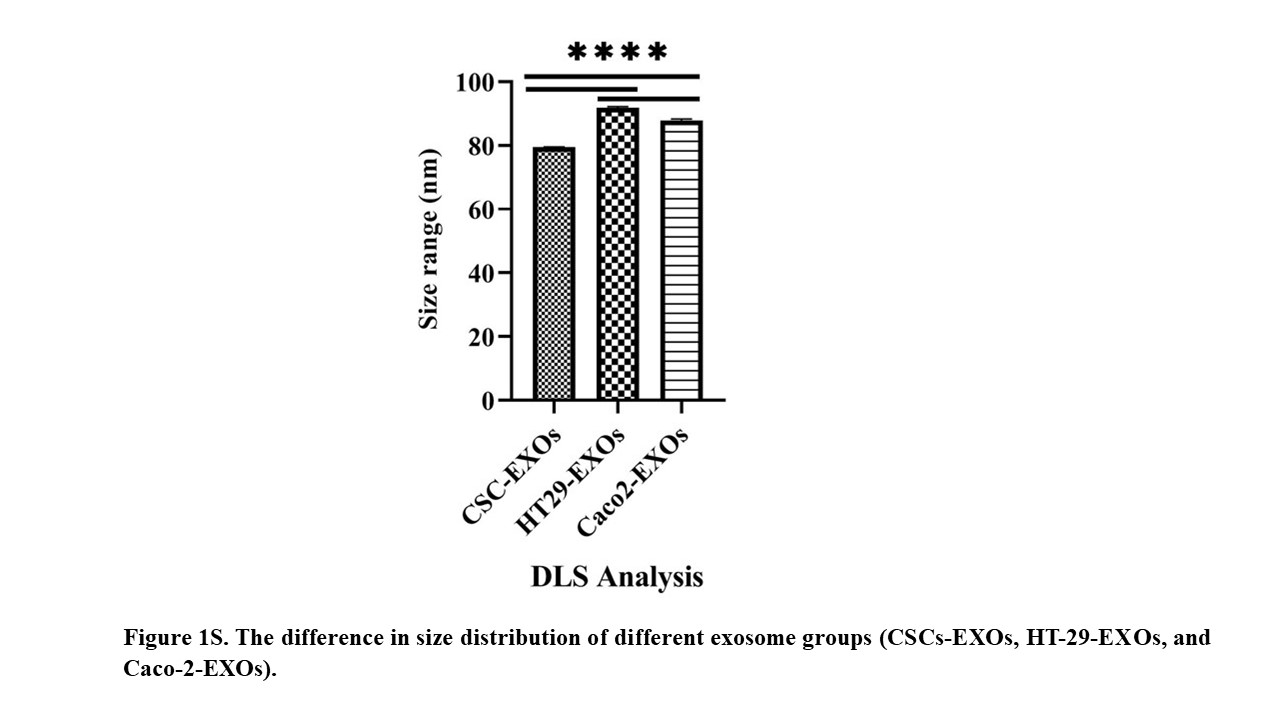

Supplement: Supplementary file 1 — Supplementary Material 1 [file 12672_2025_4295_MOESM1_ESM.jpg]
